# An Elementary Summary of Physiology

**Published:** 1818-01

**Authors:** 


*An Elementary Summary of Physiology.* By F. MAJENDIE, Doctor of Medicine of the Faculty of Paris; &c. &c.

( Continued from Vol. IV, page 486. )

CLASSIFICATION is, in some measure, arbitrary or optional; and, towards the close of our Analysis of Professor Majendie's interesting preliminary matter, we mentioned that he has found it most agreeable to his views of the human œconomy to divide the functions into three classes; namely, 1st. The Functions of Relation. 2d. The Nutritive: and 3d. The Generative. The first class comprises those functions which place the individual in relation with surrounding objects; viz. vision, hearing, smell, taste, touch, the internal sensations, the mind, voice, attitudes and motions: the detail necessarily resulting from these multifarious particulars, occupies the whole of the present volume.

We deem this the driest and least inviting portion of his work; and though, from the facts it contains, and the succinctness with which they are stated, it must prove very acceptable to the student, we apprehend there is little in it to allure the general practitioner, or the reader whose knowledge of physiology is more than elementary. To the former, therefore, we must recommend the perusal of the work itself; and for the sake of the latter, we shall give a summary view of its contents, carefully picking up in our way, and recording, any remark which bears a relation to pathology or to practice.

*Of Vision.* This Section opens with some philosophical observations on light, reflection and refraction, &c.; in them, however, we find nothing new—nothing, indeed, but what has been said—perhaps better said—by others.

There is scarcely any part of the study of nature that makes us feel so keenly the very circumscribed limits of our faculties, or tends so much to exalt physical above intellectual power, as the contemplation of the phænomena of light. The inconceivable smallness of its particles, the prodigious number of these that emanate from a luminous body during every second of time, and the velocity of their motion in a similar period, can indeed be stated in figures, but cannot be appreciated by the understanding. Thought toils after the conception in vain, and sinks hopeless and helpless under the immensity of the mere effort! To this reflection we were led by our author's remarks, as well as by the following striking sentence in an astronomical work long and deservedly popular. "If a million of the particles of light were so large as to equal in bulk an ordinary grain of sand, we durst no more open our eyes to the light, than suffer sand to be shot point blank against them."*

Dr. Majendie objects, as we think, with much reason, to the validity of the common explanation of the colour of bodies.

"Light is not homogeneous, since it is composed of very differently coloured rays, viz. red, orange, yellow, green, blue, indigo and violet. Upon this fact is founded the explanation of the colours of bodies. A white body is one which reflects light without decomposing it; a black body, one which does not reflect, but completely absorbs it. Coloured bodies decompose light, while reflecting it: they absorb part, and reflect part. Thus a body will appear green when the union of the colours which it reflects, forms green, &c. This explanation very much resembles that of the phænomena of life, by the vital properties, i. e. it explains nothing." p. 21

After the the above, there follows a neat anatomical description of the eye, and the lachrymal apparatus; which, of course, we entirely omit. He believes the conjunctiva to possess a facility of absorption, and has added a note to this effect:

"One may poison an animal by applying poisonous substances to the conjunctiva. Hence, we do not agree with Mr. Adams, a celebrated London oculist, that the extract of belladonna may be long applied to the eye without inconvenience." p. 30.

* Ferguson's Astronomy. Ed. 12th, p. 92.

This may be so; but we have never found any inconvenience result from the use of belladonna, or any other local applications to the eye; nor do we believe it a frequent, if a possible, occurrence.

Our author, with Drs. Gall and Spurzheim, traces the origin of the optic nerves not to the thalami, but to the anterior pair of the corpora quadrigemina. With regard to the conjunction of these nerves on the upper surface of the body of the sphenoid bone, he doubts much whether they decussate, or are merely in contact. Anatomy has not yet determined the question, and morbid dissections furnish proofs in favour of each of these opinions. Nevertheless he believes, that this conjunction has great influence upon the transmission to the sensorium, of impressions received by the eyes. Upon the mode of this transmission, or how the optic nerves act; as also upon the other much-agitated metaphysical question, why we judge objects erect, while their pictures on the retina are inverted, and why we see objects single with two eyes, he has wisely abstained from entering. What has foiled the sagacity of Newton, Kepler, Berkeley, Briggs, Smith, and Porterfield, is, we fear, not to be discovered as yet. Reid, in his celebrated inquiry into the human mind, took a summary method of settling these various questions, by referring them to 'Laws of Nature'; but is not this tantamount to saying, that we know nothing of the matter?

Upon another debated point in physiology, namely, the structure and motions of the iris, Dr. M. expresses himself with equal indecision. Zinn conceived this organ to be in its structure a membrane *sui generis.* M. Mery of the Academy of Sciences, and Baron Haller believed it an erectile texture, and that its motions depend upon a sympathetic influx or efflux of the fluids of its own vessels, from the action of a greater or less light on the retina. This opinion has been adopted by Blumenbach and by Richerand:* on the other hand, it has been very ably opposed by Dr. Whytt of Edinburgh,† so illustrious for his controversy with Haller. Whytt's opinion has been sanctioned by the researches of Winslow, Ruysch, the second Monro, and others,‡ who detected and de-

* "Nouveaux Elemens de Physiologie," tom. ii. p. 24.

† "Essay on the Vital and other involuntary Motions of Animals," Ed. ii. p. 140. Also, "Physiological Essays," Ed. ii. p. 144.

‡ We beg to correct the inaccuracy into which our learned author has scribed a double order of fibres in the iris, namely, a circular and a radiating; which they conceived to be muscular, and the real sphincters and laxators of the pupil.

Though our author does not inform us to which side of the question he inclines, we, for our parts, have no doubt that the most correct, as well as most recent opinion on this subject is, that the motion of the iris is owing, not to consent with the retina, but to the direct stimulus of the rays of light on its own muscular fibres. This conclusion is confirmed by the observation of some cases of amaurosis, in which the mobility of the pupil has remained unaffected, though the retina has been totally insensible: it is farther confirmed by the well-known effects of belladonna on the pupil. Surely these effects cannot be ascribed to the sympathetic influence of the retina.

Our readers will thank us for calling their attention to the following note; it indeed affords materials for reflection:

"The pupil is very large in persons debilitated by venereal excesses, in those who labour under worms, some abdominal enlargement, hydrocephalus, &c.; and after the application, for some hours, of narcotics, and especially of belladonna, to the conjunctiva; it is frequently either very large or very much contracted in cerebral affections. The motions of the pupil generally indicate the sensibility of the retina." p. 44.

On the subject of Vision, Professor M. infers from many facts, that our exact ideas of the distance, size, form, &c. of objects, are the result of practice, or of what may be called education of the sense of sight. In confirmation of this, he gives an extract from the history of the young man, blind from his birth, whom our celebrated Cheselden restored to sight by an operation for the cataract; or, as seems more probable, by incision of the membrana pupillaris. The extract affords conclusive evidence; but, though interesting in itself, it is too long for insertion here; besides, many of our readers must have seen it elsewhere.

*Of Hearing, Smell, Taste, and Touch.* These sections we must dismiss simply with the remark, that they are well

---

fallen in attributing the *discovery* of these two sets of fibres to M. Maunoir, the present Professor of Surgery at Geneva. The prior merit of having accurately described them unquestionably belongs to the eminent names we have just mentioned in the text. The Reader may also see them very well described in Dr. Porterfield's "Treatise on the Eye," Book iii. Chap. 5, p. 117.—This ingenious work was published in two volumes in 1759. Rev.

written; the anatomical descriptions concise and accurate; and the facts detailed with care, and without ostentation: but, upon the whole, they contain nothing new or very interesting.

Buffon, when speaking of the agreeable sensation produced by the approach of the sexes, said figuratively, or perhaps facetiously, that it depends upon a *sixth* sense. This casual expression was, it seems, caught up in Germany, that prolific hot-bed of hypotheses, where all manner of vagaries in religion and philosophy have from time to time been generated: and soon this pretended sixth sense was turned to good account in the reveries of magnetizers and metaphysicians. It was assigned a residence in the bones, viscera, ganglions, and nervous plexuses,—was said to be present in, and to have dominion over, all the other senses, and to be particularly developed in somnambulists and animals, giving to the former the power of predicting events, and to the latter the instinct that forewarns them of approaching dangers! Our author treats these absurdities with becoming contempt.

*Of the Internal Sensations.* These arise from the impressions which all the internal parts of our bodies have the faculty of transmitting to the brain, when they are touched by external bodies, compressed, or in any manner diseased. This is a most important province of research, and we are satisfied that when our knowledge is more advanced, when we shall be *more generally* accustomed to watch narrowly and trace closely from their commencement, the impressions produced on the centre of the nervous system, by structural or functional derangements of any organ; and when, on the other hand, we shall study with *more general* attention the effects which disorders of the brain or spinal marrow produce on the functions and sensations of individual parts, much new light will be thrown upon symptomatology in general, as well as on the various forms of mania, hypochondriasis, hydrencephalus, spasms, and the train of diseases vulgarly considered nervous and anomalous. We hoped from Professor M's undoubted learning and acuteness, that he would not have let slip so glorious an opportunity of throwing out a few pathological hints, as Newton was wont to do his luminous *queries*: but to our mortification we found, that little more than a page is allotted to the subject, and that even this little contains merely a brief, almost tabular, enumeration of such particulars as "Hunger, thirst, animal appetencies, fatigue, desire to respire, and to make water, &c!"

*Of the Brain and Nerves.* The author strongly exposes the fallacy of the successive hypotheses intended to explain the action of the nerves. He contends, that the system of the great sympathetic has no analogy with nerves, properly so called, either in its colour, form, consistence, structure, properties of tissue, or chemical properties; and, that it would be more advantageous to confess that its uses are perfectly unknown. Various twigs from it accompany the arteries of the brain; these, he thinks, have connection with the arterial parietes alone, and accompany the vessels to their ultimate divisions.

We may be allowed to insert the following remark:

"The anatomical description of the brain is one of the most perfect parts of anatomy; very recently this subject has had new light thrown upon it by the publication of the work of Drs. Gall and Spurzheim, and by the labours to which it has given rise." p. 105.

We had lately occasion to go at some length into Gall and Spurzheim's views of this organ (See Medico-Chirurg. Journal, Vol. IV.) and have only to add now, that besides the advancement of knowledge, a subordinate benefit will arise to science from their labours, inasmuch as this part of anatomy will be rid of an overwhelming load of barbarous mechanical names, borrowed from remote or imaginary resemblances to ovals, semicircles, pyramids, funnels, vaults, horns, and hoofs of hippocampi!

*Of the Intellect.* "Whatever may be the number and diversity of the phænomena which belong to the human intellect, however different they may appear from all other phænomena of life, and however evidently they may depend upon the soul, it is necessary to consider them as the result of the action of the brain, and not in any measure to distinguish them from other phænomena which depend upon organic action. In truth, the functions of the brain are absolutely subjected to the same general laws as the other functions; are developed and deteriorated with the progress of age; are modified by habit, sex, temperament, and individual disposition; are troubled, weakened or exalted in disease; perverted or destroyed by physical lesions of the brain; and, lastly, like all other organic actions, are not susceptible of any explanation; and to study them, we must confine ourselves to observation and experiment, divesting ourselves of every hypothetical idea." p. 115.

Now, we certainly think that, without the brain as their medium, the intellectual faculties cannot display themselves: yet we are very reluctant (however consonant it may be to analogy with other parts of the physiology of our frame,) to agree that the mind should be considered as in itself nothing more than a mere function of matter; since, so far as we can judge, such views inevitably lay prostrate its imperishable honours, and annihilate the strongest arguments, deduced from reason, in favour of its immortality; leading ultimately to a slightly-modified materialism; that chilly doctrine, unfavourable alike to the best interests and best hopes of mankind! Let us not deceive ourselves with the sophism, that 'the important belief of the mind's immortality is sufficiently secured by the Doctrines of Revelation, even though reason could adduce no presumption in its favour'; for it will be found, that the former have but imperfect sway on the conviction of mankind, unless reason go along with them as far as it can, and lend them, at all points, its sanction, concurrence and support. Faith, indeed, must fundamentally lean upon reason, else it is an empty sound to say, 'we believe'!

While physiologists study man in his *animal*, they ought never to forget his *intellectual* nature, which is immaterial and immortal, like *His* who gave it. We are ever sorry to see opinions, (and such opinions are fashionable at present!) held forth that tend to depreciate this noblest characteristic of the human race, and degrade it from an active primary *ens*, to a something subservient and subsidiary to organization; from an original and presiding principle, to a very result—a mere abstraction! For our parts, (with a philosophical writer of no mean note or acuteness) we prefer the "scheme which never forgets Deity, but postpones every thing *corporeal* to the *primary mental cause*. 'Tis here it looks for the origin of *intelligible* ideas, even of those which exist in human capacities. For though *sensible* objects may be the destined medium to awaken the dormant energies of man's understanding, yet are those energies themselves no more contained in *sense*, than the explosion of a cannon in the spark which gave it fire." *

Besides these weighty objections to the mind being

---

* Harris's Hermes, Ed. 2d. p. 392, &c.

Should our readers wish to see these views of the mind more ably and fully illustrated, we refer them to 'Cudworth's Intellectual System,' and to the chapter 'On a Future Life,' in 'The Analogy of Religion' by Bishop Butler, one of the most distinguished names of the Anglo-Augustan Age. He treats this abstruse subject with admirable accuracy, profoundness, closeness, and acuteness of reasoning. Rev.

considered a mere function of the brain, in like manner as digestion is a function of the stomach, or secretion of the liver, we are not over and above satisfied with the learned Professor's division of the intellectual powers into sensibility, memory, judgement, desire or will, instinct, and passions. But we shall neither state our objections, nor embody in our pages his various remarks, as we have no wish to entangle our readers in the meshes of controversy, or to benight them in the penumbra of metaphysics.

*Of the Voice.* Since the voice results from hearing and an intellectual operation, it cannot be developed in infants deaf from birth, or in idiots who are unable to establish any relation between the words they hear, and those which their larynx can produce. This holds, even though the vocal apparatus be ever so perfect.

The change in the voice's tone at puberty, the author considers owing to "greater activity of nutrition" taking place, and causing an increase in the volume of the muscles and cartilages of the larynx. The human voice possesses the conjoint powers of a wind and a stringed instrument. By the division of both recurrent nerves it is wholly lost; if one only is cut, it is half lost; this effect is owing to paralysis of the thyro-arytenoid muscles. The author has verified these results by experiments on animals.

We do not remember ever to have seen the curious art of ventriloquism so well explained as in the following passage:

"The foundation of this art is easily understood. We have instinctively observed, that sounds alter from many causes; that, for instance, they become weak, less distinct, and change their note in proportion to their distance. If a man goes to the bottom of a well, and wishes to speak to persons at its edge above, they will hear his voice modified by the distance and form of the canal which it has passed through. If, therefore, a person attentively remarks these modifications, and endeavours to produce them, he will produce an acoustic illusion, which cannot be avoided any more than we can avoid seeing objects larger when we look through a magnifying glass; the error will be perfect, if tricks are at the same time played to distract our attention. The ventriloquist's voice is formed in the ordinary way: he merely modifies its volume at pleasure; and if he happens to speak without moving his lips, it is because he takes care to employ words in which there are no labial consonants. In a certain sense we may say, that his art is to the ear, what painting is to the eye." p. 159.

After this, it must be obvious that *ventri*-loquism is a ridiculous term for this art.

*Of Muscular Contraction, Attitudes and Motions.* We merely *notice* these sections, as they evince Dr. M. to be a very shrewd, as well as a very patient, observer: but they contain nothing for our Readers' purpose. We pass them over the more willingly, as our pages have been so recently occupied with the same subject.*

The following extract, however, is well deserving of attention:

"We may affirm, that the determining cause of motion is the will; but the production of the muscular contraction, necessary for motion, does not depend upon the will; it is purely instinctive. For instance, I can move my arm or hand, but am unable to contract the muscles of these parts separately or altogether, unless I have an idea of the motion to be produced.

"After these considerations, it would be right to conclude that will, and the action of the brain which directly produce the contraction of muscles, are two distinct phænomena; but the direct experiments of modern physiologists, and particularly of Le Gallois, have put this truth in the strongest light. His experiments have demonstrated, that the will is more particularly seated in the cerebrum and cerebellum. The direct cause of motion appears, on the contrary, seated in the spinal marrow. If the spinal marrow is separated from the rest of the brain, by a section behind the occipital bone, the will is prevented from determining and directing the motions, but the latter are not the less produced. It is true, that as soon as the separation is made, they become very irregular in extent, rapidity, duration, and direction." p. 209.

We here close our Analysis of the First Volume of Professor Majendie's Work. From the nature of its contents, it is necessarily the least interesting of the two. The execution of it is such as will not detract any thing from the well-merited reputation of the author; nor, on the other hand, do we think that it will add any thing thereto, as the new facts he has supplied are entirely of a subordinate kind. Upon the whole, speaking of the volume before us, the work may be useful enough to students, but we cannot see how it has deserved the eulogium passed upon it by the Gentleman who has given it an English dress. He, indeed, has characterized it as "a most admirable production, infinitely superior to every other

---

* Medico-Chirurg. Journal, (for November 1817.) Vol. iv. p. 430.

work upon physiology, except perhaps Blumenbach's Institutions." Now we would put it to the Gentleman, whether he has not, in the height of his enthusiasm, overlooked or forgotten the "Conspectus Medicinæ Theoreticæ", a work both in matter and style every way worthy of the modern Cicero of medical literature: but, upon reflection, we are disposed to overlook this unmeasured extravaganza, and to make large allowance for the sympathetic bias of a translator towards his author; a bias, which is the tenderest of literary ties, excepting always the parental and exclusive fondness of a writer for *his own* cerebral offspring!

The translation, nevertheless, is executed with fidelity and ability. We only noticed one mistake throughout: at page 34, *posterior* is put for *anterior;* this can be very easily corrected. In other respects, the volume enjoys a very creditable exemption from typographical blunders.

P. S. Since writing the above we have received, direct from Paris, the second and last volume of Dr. Majendie's work. We shall analyse it from the original, and present our readers with a summary of its contents, in an early number. It is by far the most valuable and interesting volume of the two. REV.

---

